# Structural and functional diversity of caspase homologues in non-metazoan organisms

**DOI:** 10.1007/s00709-017-1145-5

**Published:** 2017-07-25

**Authors:** Marina Klemenčič, Christiane Funk

**Affiliations:** 1 Department of Chemistry, Umeå University, 901 87 Umeå, Sweden; 2 Department of Chemistry and Biochemistry, Faculty of Chemistry and Chemical Technology, University of Ljubljana, Večna pot 113, 1000 Ljubljana, Slovenia

**Keywords:** Algae, Cyanobacteria, Cell death, Cysteine protease, Metacaspase, Orthocaspase

## Abstract

Caspases, the proteases involved in initiation and execution of metazoan programmed cell death, are only present in animals, while their structural homologues can be found in all domains of life, spanning from simple prokaryotes (orthocaspases) to yeast and plants (metacaspases). All members of this wide protease family contain the p20 domain, which harbours the catalytic dyad formed by the two amino acid residues, histidine and cysteine. Despite the high structural similarity of the p20 domain, metacaspases and orthocaspases were found to exhibit different substrate specificities than caspases. While the former cleave their substrates after basic amino acid residues, the latter accommodate substrates with negative charge. This observation is crucial for the re-evaluation of non-metazoan caspase homologues being involved in processes of programmed cell death. In this review, we analyse the structural diversity of enzymes containing the p20 domain, with focus on the orthocaspases, and summarise recent advances in research of orthocaspases and metacaspases of cyanobacteria, algae and higher plants. Although caspase homologues were initially proposed to be involved in execution of cell death, accumulating evidence supports the role of metacaspases and orthocaspases as important contributors to cell homeostasis during normal physiological conditions or cell differentiation and ageing.

**Electronic supplementary material:**

The online version of this article (doi:10.1007/s00709-017-1145-5) contains supplementary material, which is available to authorized users.

## Introduction

“Out of life’s school of war: What does not destroy me, makes me stronger.” wrote the German philosopher Friedrich Nietzsche in his book *Twilight of the Idols or how to philosophize with a hammer*. Even though reformatted to more common use, this phrase has been used to describe the dual nature of caspase homologues (Hill and Nystrom 2015), portraying the importance of this class of proteins in metabolic processes of living and dying. We will, herein again, use this catch phrase, to present recent advances in research on proteins belonging to the C14 family of cysteine proteases in non-metazoan organisms and will focus on cyanobacteria, algae and higher plants. We will compare the structural and functional properties of metacaspases and metacaspase-like proteases to their homologous aspartate-directed caspases; the latter are known to be involved in the initiation and execution of apoptosis, the prominent form of programmed cell death in Metazoa. However, we neither aim to discuss definitions of programmed cell death nor to contribute to the ongoing debate about the evolution of the cell death machinery. Instead, this review will summarise data on metacaspases, metacaspase-like proteases and orthocaspases with focus on chlorophyll-containing organisms.

## Family C14

**Fig. 1:**
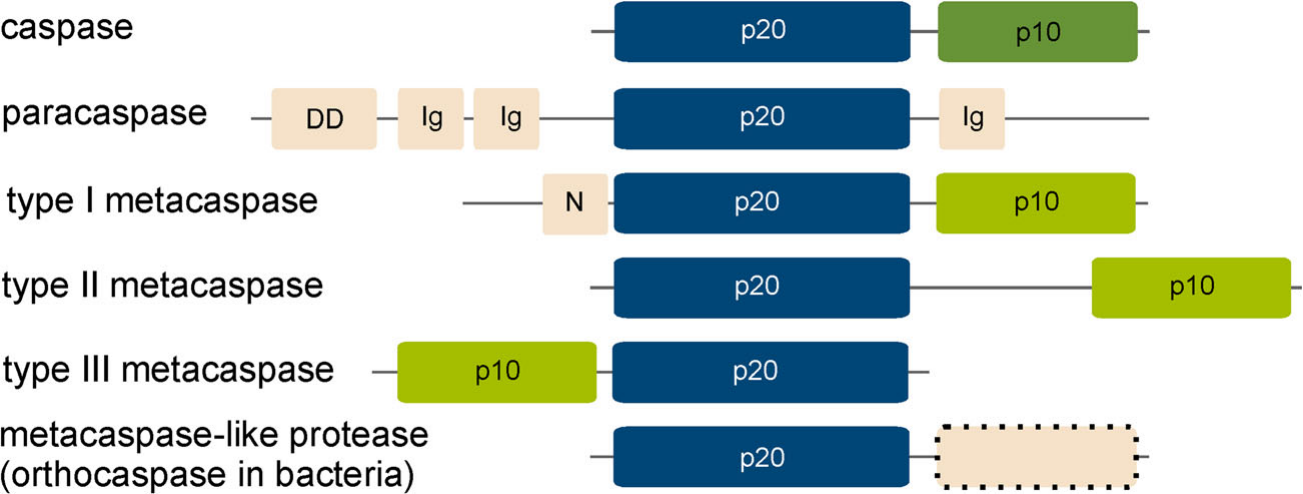
Schematic domain organisation of the C14 cysteine proteases. Domains were identified using InterPro protein sequence analysis and classification tool. The catalytic p20-like domain is coloured in *dark blue* and the p10 domain in *green*; *light green* indicates the presence of a 280-loop involved in calcium binding found in metacaspases. Additional domains are coloured in *light red*. A *dashed border* indicates the presence or absence of additional domains. Figure is not drawn to scale. *Ig* immunoglobulin-like domain, *DD* death domain, *N* N-terminal proline-rich repeat

**Fig. 2:**
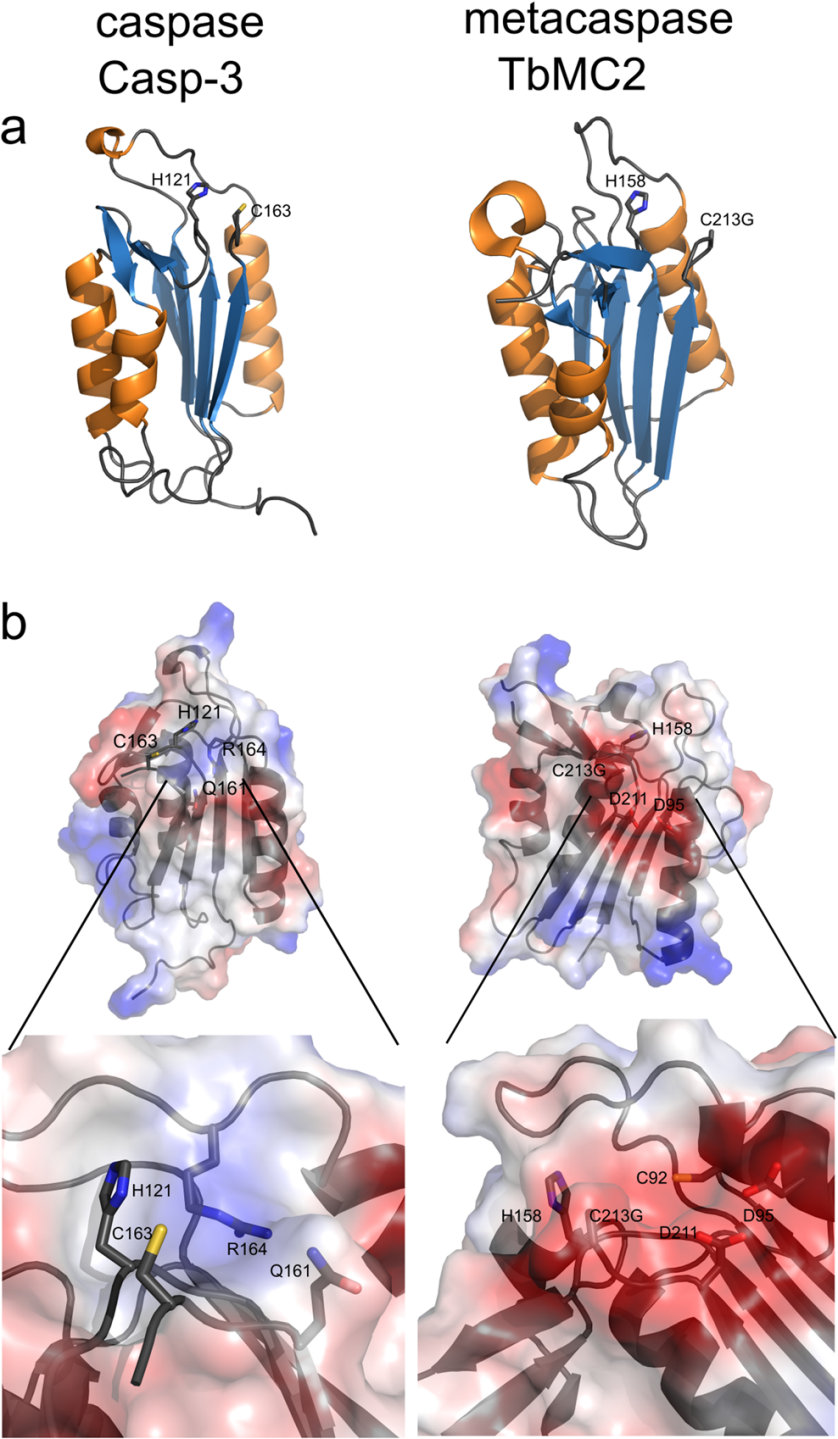
Comparison of the properties of p20-fold and specificity pocket in caspases and metacaspases. The p20 domain of caspase-3 (Casp-3), PDB ID: 3gjt (Fang et al. 2009) is compared with the type I metacaspase TbMC2, PDB ID: 4af8 (McLuskey et al. 2012). **a** Ribbon representation of the p20 domains: α-helices are coloured in *orange* and β-sheets in *blue*, side chains of the amino acid residues of the catalytic dyad are shown as *sticks*. **b** Surface potentials of caspase-3 and TbMC2; *blue* indicates basic amino acids, *red* acidic amino acids. The *inlets* display the specificity pockets in more detail. Side chains of the amino acids in the catalytic dyad and specificity pocket are shown as *sticks*

The CD clan C14 of the MEROPS peptidase database, containing caspases and their homologues: the metacaspases, metacaspase-like proteases/orthocaspases and paracaspases, is the most ubiquitous of all CD families, with representatives in all kingdoms of life. Throughout this review, we will follow the classification in MEROPS, where the terms caspase-like proteins or caspase homologues is limited to their structural homologues, i.e. all the proteins containing the p20 domain (Fig. 1). Caspases are found in animals and a few viruses, paracaspases in the genomes of slime mould and metazoa and metacaspases in organisms ranging from simple prokaryotes to higher plants, but they are absent in animals. Similar to all members of the CD clan, members of the C14 family hydrolyse the peptide bonds of their substrates using a catalytic dyad consisting of cysteine and histidine (McLuskey and Mottram 2015), which is situated within a characteristic caspase/haemoglobinase fold (Aravind and Koonin 2002) composed of four β-sheet strands and three α-helices (Fig. 2a). The name of this so-called p20 domain is derived from caspases (Walker et al. 1994), which are synthesised as inactive procaspases comprised of a prodomain, a large catalytic domain of approximately 20 kDa (p20) and a small regulatory domain of approximately 10 kDa (p10). Removal of the prodomain induces autocatalytic cleavage within an inter-domain linker region between the p20 and p10 domains, generating active caspase dimers. While all members of the C14 family contain the catalytic p20 domain, the small p10 domain is only found in metacaspases and caspases. We would like to point out that paracaspases do *not* contain p10 domains homologous to either caspases or metacaspases (Choi and Berges 2013). Paracaspases were suggested to be classified into two groups: type I paracaspases containing the p20 domain, a death domain (DD) and immunoglobulin domains (Ig), as found in the best characterised paracaspase, MALT-1 (Yu et al. 2011) (see also Fig. 1), and type II paracaspases, containing only the caspase p20 domain (Hulpiau et al. 2016). Distinction between metacaspases and paracaspases was proposed also to be based on the motive surrounding the catalytic cysteine residue (DxCH for metacaspases and DxCR for proposed type II paracaspases). However, while the DxCR motif is characteristic for caspases and paracaspases, it also is found in many prokaryotic caspase homologues, which undisputedly are classified as metacaspases and not paracaspases. Additionally, based on phylogenetic analyses, prokaryotic caspase homologues containing only the p20 domain are not grouped with paracaspases regardless of their catalytic Cys motif (Tsiatsiani et al. 2011). We therefore prefer to use the term “metacaspase-like proteases” for non-metazoan caspase homologues lacking the p10 domain as suggested by (Choi and Berges 2013). Notably, not all putative metacaspase-like proteins contain a catalytic cysteine-histidine dyad: in 16% of the analysed putative metacaspase-like sequences, the histidine residue was substituted by a polar serine and the catalytic serine by a hydrophobic tyrosine (Asplund-Samuelsson et al. 2012) and therefore might be catalytic inactive. Recently, prokaryotic metacaspase-like proteases were termed orthocaspases. However, only one orthocaspase, MaOC1 from *Microcystis aeruginosa* PCC 7806 (Klemencic et al. 2015), has been biochemically characterised up to now.

## Classification and structure

### Caspases

To be able to compare metacaspases and metacaspase-like proteases of photosynthetic organisms, their relatives in animals have shortly to be described. Caspases are synthesised as inactive zymogens that are autocatalytically processed at specific aspartic acid residues between the p20 and p10 domains. The two domains of a caspase monomer, the p20 and p10 domain, are folded into a central six-stranded β-sheet with a highly conserved cleavage site. In an active enzyme, dimers are formed via the two β-sheets of the p10 domain. The p10 domain therefore is not only important for dimerization, but also contains amino acid residues actively involved in catalysis (Salvesen et al. 2016). Caspases are divided into two groups: inflammatory and apoptotic caspases, with the latter further organised into initiator and executioner caspases. Executioner caspases contain short prodomains of approximately 25 amino acid residues required for their dimerization, while the inflammatory as well as the initiator caspases possess at their N-termini large prodomains of approximately 100–200 residues. Both, inflammatory and initiator caspases, can contain CARD (caspase recruitment domains), while DED (death effector domain) motifs can only be found in initiator caspases (MacKenzie and Clark 2012). Despite their diverse N-terminal regions, the catalytic domain of caspases, the p20 domain, has a highly conserved fold in all crystal structures determined to date and therefore has been used as a stencil to identify members of the C14 family (McLuskey and Mottram 2015) (Fig. 2a). In addition to the catalytic His and Cys residues, caspases contain a basic specificity pocket, consisting of highly conserved positively charged residues (Gln161 and Arg164 in caspase-3, Fig. 2b), explaining their Asp-P1 preference. Peculiarly, caspases are the only members of the C14 family cleaving their substrates after negatively charged amino acid residues.

### Metacaspases

In contrast to caspases, all remaining members of the protease C14 family characterised up to now exhibit a strict preference for substrates containing basic arginine and/or lysine residues at the P1 position, which is a consequence of an acidic specificity pocket (Fig. 2b). Among these, metacaspases represent the largest sub-family and are further classified into three types according to their architecture of the p20 and the p10 domains (Fig. 1). Type I metacaspases can contain an additional proline-rich repeat and zinc-finger motif in the N-terminal prodomain, whereas type II metacaspases lack additional N-terminal motives and are hallmarked by the presence of an extended linker region between p20-like and p10-like domains (Vercammen et al. 2004). While type I metacaspases can be found in lineages from *Proteobacteria* to plants, type II metacaspases are exclusively found in the green lineage of plants and algae. Recently, genes encoding type III metacaspases have been identified only in algae that arose after secondary endosymbiosis. These proteases contain an unusual rearrangement of domains, with the p10-like domain located N-terminal instead of C-terminal as in other members of the caspase family (Choi and Berges 2013). As opposed to caspases, metacaspases do not undergo dimerization for their activation. Instead, the activity of all three metacaspase types strongly depends on the presence of calcium ions (Moss et al. 2007; Wong et al. 2012); the only exception seems to be *Arabidopsis thaliana* type II metacaspase, AtMC9, whose activity was shown to be calcium-independent (Zhang and Lam 2011). In type II metacaspases, presence of CaCl_2_ in millimolar concentrations induces specific cleavage in the linker region connecting the p20 and p10 domains, similar to the activation observed in caspases (Lam and Zhang 2012; Piszczek et al. 2012; Vercammen et al. 2004). For type I metacaspases, auto-processing never occurs between the p20 and p10 domains: TbMC2 from *Trypanosoma brucei* underwent non-specific in vitro cleavages, when isolated at high protein concentrations (Moss et al. 2007), while the N-terminal domain of *A*. *thaliana* metacaspase AtMC1 in vivo was cleaved and completely removed (Coll et al. 2014; Coll et al. 2010). Recently, we were the first to biochemically characterise the type III metacaspase GtMC2, from the cryptophyte *Guillardia theta* (Klemencic and Funk 2017). Our data confirm the close relation of type III and type I metacaspases, as suggested by Choi and Berges (2013). No cleavage was observed in the recombinant GtMC2 full-length protein; however, calcium-dependent removal of the N-terminal domain clearly resulted in proteolytic activity; this process was shown to require mM concentration of calcium ions (Klemencic and Funk 2017). Two distinct calcium binding sites were identified in type I and type III metacaspases with different binding affinities: one in the low micromolar and the second in the low millimolar range (Klemencic and Funk 2017; Machado et al. 2013). While the high-affinity binding site can be undoubtedly located on the p20 domain, our results suggest the location of the low-affinity binding site in the p10 domain, more precisely in the so-called 280-loop (McLuskey et al. 2012). The negatively charged residues in the p10 domain are highly conserved in all three types of metacaspases. We therefore propose their general involvement in binding of calcium ions at high micromolar concentrations, thus controlling the catalytic mechanism in all three metacaspase types. Lack of the p10 domain might therefore at the same time explain the calcium-independent activation of paracaspases and orthocaspases, both p10-less members of the C14 family.

### Metacaspase-like proteases and orthocaspases

**Fig. 3:**
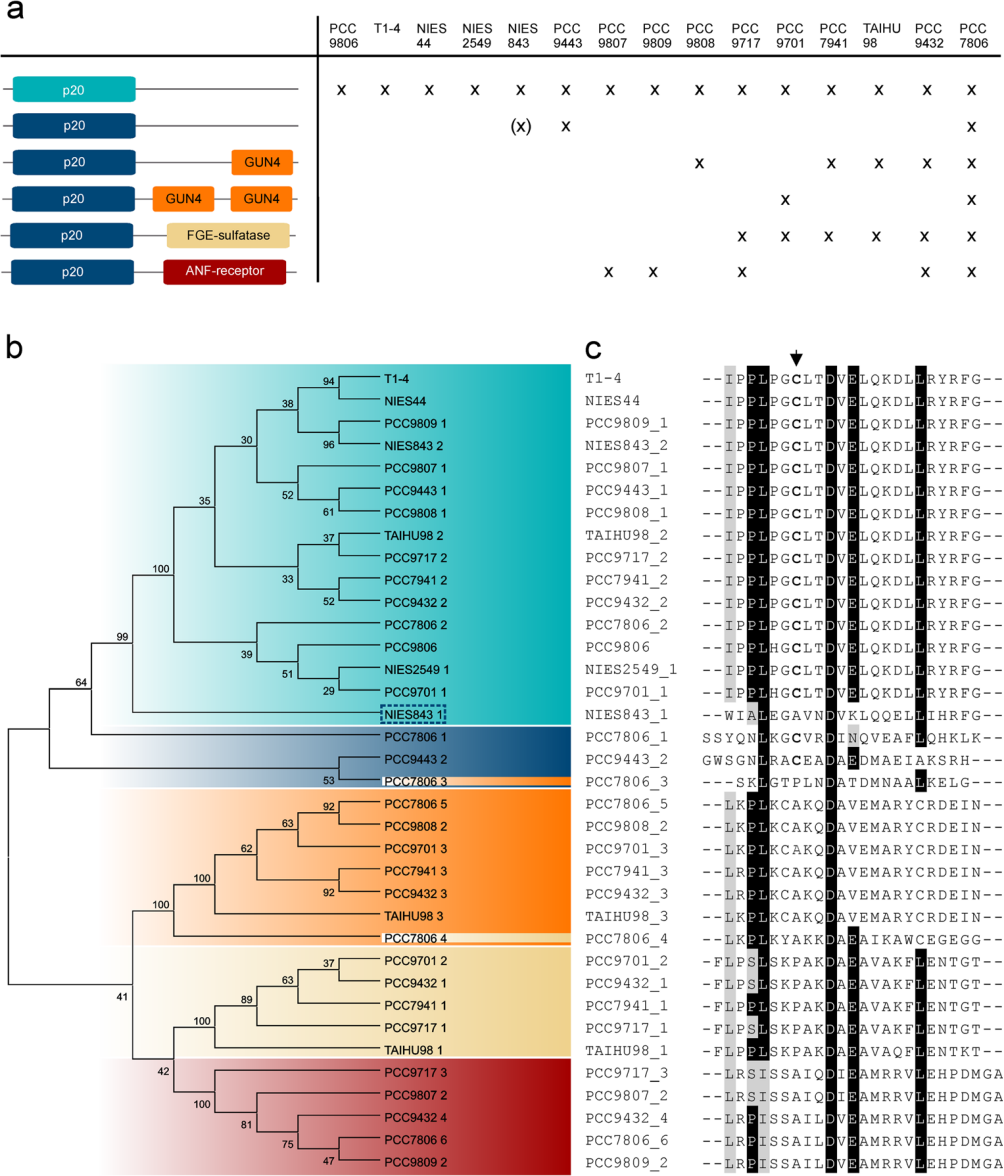
Overview of orthocaspases in various *Microcystis aeruginosa* strains and phylogenetic analysis of their p20 domains. **a** Overview of orthocaspases in all *M. aeruginosa* strains sequenced up to now. Strain and domain names can be found in Supplemental table (Online resource 1). Figure is not drawn to scale. The orthocaspase NIES-843 of *M. aeruginosa* is written in *brackets* to indicate its mutation of the cysteine residue in the specificity pocket (see also (**c**)). **b** Unrooted phylogenetic tree of all identified *M. aeruginosa* orthocaspases using the Neighbour-Joining method of MEGA software (version 6), based on their p20-like sequences aligned by PROMALS. NCBI identifiers can be found in Supplemental table (Online resource 1). Bootstrap values are shown on *branches* as percentages. *Colours* represent the active (*dark blue*) or putatively inactive (*light blue*) catalytic domain or the presence of additional domains C-terminal to the p20 domain with the same colour code as in **a**. **c** Sequence alignment of the specificity pocket in the region surrounding the cysteine residue. Identical residues are coloured *black* and similar amino acids are *shaded* in *grey* with 70% threshold for colouring. Conserved cysteine residues in the specificity pocket are marked in *bold*, their position is indicated by an *arrow*. The sequence alignment was performed using PROMALS and the figure was generated using BioEdit

Despite growing interest in research of plant and algal metacaspases, proteases containing only the p20 domain remain the most neglected members of the C14 family. These caspase homologues, lacking the p10 domain, are found in prokaryotic (orthocaspases) as well as eukaryotic (metacaspase-like proteases) organisms. However, only orthocaspases can contain a variety of additional domains, commonly located C-terminal to the putative catalytic p20 domain (Asplund-Samuelsson et al. 2012). Interestingly, during evolution, the structure of metacaspase-like proteases seems to have been simplified: in early metazoan animals as well as all up to now characterised algae emerging from primary or secondary endosymbiosis, they consist of only the p20 domain (Choi and Berges 2013; Hulpiau et al. 2016). Among bacteria, strains belonging to α-proteobacteria, δ-proteobacteria and cyanobacteria are especially rich in the number of putative caspase homologues (Asplund-Samuelsson et al. 2012). The more complex filamentous diazotrophic cyanobacteria contain a large number of orthocaspase genes: 12 genes are present in *Trichodesmium erythraeum* IMS 101, 9 in *Anabaena variabilis* ATCC 29413 and 9 in *Nostoc punctiforme* (Jiang et al. 2010). The number of orthocaspase genes varies not only from species to species, but can differ considerably within one species as well. Such example are strains of the unicellular *M*. *aeruginosa*, of which 15 genomes were sequenced up to now (Frangeul et al. 2008; Humbert et al. 2013; Kaneko et al. 2007; Okano et al. 2015; Yamaguchi et al. 2016; Yamaguchi et al. 2015). In this species, the number of orthocaspase genes ranges from one (e.g. *M*. *aeruginosa* PCC 9806, *M*. *aeruginosa* sp. T1-4, Fig. 3a), containing only the catalytic p20 domain, to six orthocaspase genes (*M*. *aeruginosa* PCC 7806), termed *MaOC1-MaOC6* (Klemencic et al. 2015). Interestingly, all *M*. *aeruginosa* strains contain at least a putatively inactive variant with substitutions in the active site (Fig. 3a). Phylogenetic analysis, based solely on their p20 domains, shows clear separation among proteolytic active and inactive enzymes based on their conservation of the His-Cys dyad and absence or presence of additional domains within the p20-bearing polypeptide chain (Fig. 3b). A peculiar exception are the orthocaspases of the strain *M*. *aeruginosa* PCC 7806, where phylogenetic analysis clusters, e.g. p20 domain of MaOC4, which also contains a FGE-sulfatase domain, together with the p20 domains harbouring additional GUN4 domains, suggesting domain-swapping within the strain. The domains of the *M*. *aeruginosa* PCC 7806 orthocaspases are highly variable: MaOC3 and MaOC5 were found to contain sequences homologous to the GUN4 domain, while a sequence homologue to the sulfatase-modifying factor enzyme 1 is present at the C-terminus of MaOC4. MaOC6 is most likely a transmembrane protein with an extracellular or periplasmic receptor domain at the C-terminus. However, the variety of the domains within one species seems to be conserved, i.e. the *M*. *aeruginosa* strains lack other domains, which can usually be found in cyanobacteria. In other cyanobacteria, domains that have been linked to protein-protein interactions (WD40 domain, tetratricopeptide repeat domain; TPR_1, TPR_2 and Sel1), signal transduction (ANF_receptor, an extracellular ligand-binding domain, GGDEF, a domain synthesising intracellular signalling molecule cyclic di-GMP and CHASE2, a bacterial extracellular receptor domain), and/or domains that have been linked to eukaryotic PCD such as NACHT domain, harbouring a predicted nucleoside-triphosphatase (NTPase) domain (Asplund-Samuelsson et al. 2012) can be found. *Gloeobacter violaceus* PCC 7421, an early-branching cyanobacterial strain (Nelissen et al. 1995), possesses five genes, which beside the conserved p20 domain are rich in WD40-repeat-containing domains, pointing again to the importance of additional domains in prokaryotic orthocaspases. It should also be noted that not all prokaryotes, neither all cyanobacteria, are equipped with orthocaspase genes. *Bacillus subtilis* as well as *Escherichia coli* are lacking these proteases, as do the unicellular non-nitrogen-fixing marine strains of the genera *Synechococcus*, *Prochlorococcus*, *Cyanobium* and *Cyanothece* (Asplund-Samuelsson et al. 2012). The complexity of prokaryotic p20-containing proteins and especially their simplification in evolutionary more advanced organisms makes research in this field truly exciting.

## Function

### Metacaspases in higher plants

Less data are available from plants than those from Metazoa explaining the cell death processes, still, the topic has been discussed extensively in recent reviews, which are recommended to the reader (Fagundes et al. 2015; Minina et al. 2017; Salvesen et al. 2016; Sueldo and van der Hoorn 2017). In this review, we therefore only will give a general overview of metacaspases and their roles in cell death and other processes in plants.

The current classification separates programmed cell death (PCD) processes in plants in three distinctive categories based on their morphological and biochemical features: (i) vacuolar cell death, (ii) necrosis and (iii) mixed (van Doorn and Woltering 2005). Vacuolar cell death is characterised by the growth of lytic vacuoles, which, by gradually increasing in size, engulf the cytoplasmic content and finally burst. The resulting acidification of the cell and the release of proteolytic enzymes then leads to self-clearance of the dying cells (Minina et al. 2014). In contrast, acute stress triggers necrosis, which is marked by mitochondrial dysfunction, uncontrolled rupture of the plasma membrane and disordered cell lysis (Minina et al. 2013). Since plant cells are surrounded by rigid cell walls instead of cytosolic swelling, observed in animal necrosis, the cells shrink. Mixed cell death is characteristic for the hypersensitive response (HR), which occurs locally to defend against pathogens and therefore morphologically resembles necrotic cell death. However, these features are often accompanied by the phenotype of vacuolar cell death (van Doorn et al. 2011). While vacuolar cell death is indispensable for physiological plant development, necrosis usually occurs as response to abiotic stress or pathogen attack (van Doorn et al. 2011). Vacuolar and necrotic programmed cell deaths therefore are observed not only in differed phenotypes, but as a consequence to distinct physiological conditions, i.e. during development or as stress response.

Metacaspases have been identified to be active during vacuolar and nectrotic PCD. Type II metacaspases have been associated with developmental PCD in *A*. *thaliana* (AtMC9) and Norway spruce (McII-Pa). AtMC9 was shown to be involved in the post-mortem autolysis of xylem (Bollhoner et al. 2013), a crucial process for xylem formation, where the cytosolic content is removed from dead xylem vessel cells to allow unobstructed water transport. In fact, *AtMC9* was shown to be the only *Arabidopsis* metacaspase gene strongly upregulated during xylem and also during lateral root cap development (Olvera-Carrillo et al. 2015). Furthermore, in *Arabidopsis* cell cultures AtMC9 was shown to suppress the development of tracheary elements; down-regulation of *AtMC9* transcription induced autophagy (Escamez et al. 2016). Contrary, in spruce embryo suspensor cells, McII-Pa was shown to promote autophagy (Minina et al. 2013). Induction of autophagy was shown to depend on functional McII-Pa and important for the route of cell death, vacuolar or necrotic. The contradicting results from *Arabidopsis* and Norway spruce reflect the complexity of autophagy, which is observed in various cellular processes.

Type I as well as type II metacaspases have been shown to be involved in stress responses and HR, thus exhibiting necrotic and mixed phenotypic cell deaths. Transcription of genes coding for the type II metacaspases AtMC4 and AtMC5 was upregulated during infection with bacterial pathogens (Watanabe and Lam 2005); additionally, AtMC4 expression was increased during abiotic and biotic stress (Watanabe and Lam 2011a). Even the type II metacaspase AtMC8 was shown to positively respond to abiotic stress, mediating UV—as well as hydrogen peroxide-induced cell death (He et al. 2008). Of two highly homologous type I metacaspases AtMC1 and AtMC2, AtMC1 was shown to act as positive regulator of cell death, while AtMC2 seems to be its antagonist, inhibiting activity of AtMC1. Direct interaction of these two proteins was excluded, their mode of action thus remains to be clarified (Watanabe and Lam 2005). Increasing evidence suggests that plant metacaspases are involved not only in death-related events, but also are important for survival of the plant cell. In ageing plants, the type I metacaspase AtMC1 seems to participate in the removal of age-related cell aggregates (Coll et al. 2014). The dual role of AtMC1 on one side inducing cell death and on the other side acting as survival factor again indicates the delicate fine tuning of cellular processes orchestrating actions from various external and internal stimuli.

### Metacaspases and metacaspase-like proteases in (micro)algae

Studies of algal PCD have high economic importance: the rapid collapse, which is unrelated to grazing or sedimentation, but a cellular response to various abiotic and/or biotic stressors, is highly desired in algal blooms, but devastating for industrial cultures. The initial assumption that microbial populations consist of competing and selfish individuals, driven by their own need of existence, needed to be reevaluated. Active cell death in unicellular organisms can be seen as an altruistic mechanism conferring the survival of the remaining cells and increasing the genetic fitness of the population as a whole. The evolution of PCD in microorganisms has been comprehensively reviewed elsewhere (Bidle 2016; Bidle and Falkowski 2004; Durand et al. 2016; Franklin et al. 2006) and is beyond the scope of this paper. Many unicellular organisms are reported to undergo PCD with characteristic morphological and biochemical changes (as reviewed by Berman-Frank et al. 2004), accompanied by the activation of “metacaspases”, which was assayed either by measuring specific enzymatic activity using classical tetrapeptide caspase substrates (DEVD, IETD, VDVAD etc.) or by immunodetection using various antibodies derived from mammalian caspase antisera. Due to the high specificity of the caspase-hemoglobinase fold, enhanced immunohybridization signals using anti-caspase antibodies might de facto reflect increased protein levels of metacaspases. However, as discussed earlier, metacaspases exhibit no activity towards classical caspase substrates, therefore substrates with positively charged Arg or Lys residues at P1 position should be used to indicate metacaspase activity in these organisms (Tsiatsiani et al. 2011). Nevertheless, increased caspase activity (activity towards substrates with Asp residues at P1 position) frequently coincided with other biochemical markers characteristic for PCD, suggesting either activation of proteases with Asp-activities downstream of metacaspases or increased total cellular metabolic activity upon induction of stress, resulting in increased expression of diverse proteolytic enzymes. Increased overall proteolytic activity was indeed observed and reported in one of the first studies exploring the cell death of phytoplankton by measuring hydrolysis of leucine β-naphthylamide. Upon nitrogen starvation, a 12-fold increase of non-specific proteolytic activity was observed in the diatom *Thalassiosira weissflogii* and a 4-fold increase in the chlorophyte *Dunaliella tertiolecta* (Berges and Falkowski 1998). Similar data have been obtained of the filamentous freshwater cyanobacterium *Anabaena sp*., where exposure to salt stress resulted in increased non-specific proteolytic activity (Ning et al. 2002).

The first specific involvement of cysteine proteases in PCD was observed when the classical cysteine protease inhibitor E-64 suppressed autolysis in the secondary endosymbiont, the dinoflagellate *Peridinium gatunese*, exposed to inorganic carbon (CO_2_) stress (Vardi et al. 1999). The only metacaspase gene of another dinoflagellate, *Prorocentrum donghaiense*, was significantly higher expressed after phosphate depletion compared to non-treated cultures. The maximal expression was observed after 11 days of depletion (Huang et al. 2016). Interestingly, this abiotic stress also induced elevated protease activity towards VAD substrate, with maximal activity after 13 days; the metacaspase therefore could activate downstream proteases with caspase-like activity. Even in diatoms, relatives to dinoflagellate, iron starvation and culture age were shown to activate metacaspases. The six metacaspases (TpMC1-TpMC6) of the diatom *Thalassiosira pseudonana* exhibit distinct expression patterns on RNA- (RT-qPCR) and protein-level (immunolabelling). Elevated gene and protein expression of the two metacaspases (TpMC2 and TpMC4) were reported during the late culture phase, accompanied by markers for photosynthetic stress and PCD. However, high and constitutive gene- and protein- expressions of TpMC1, TpMC3, TpMC5 and TpMC6 were observed in living *T*. *pseudonana* cells, but decreased during physiological stress and death (Bidle and Bender 2008). When *T*. *pseudonana* cells were exposed to the polycyclic aromatic hydrocarbon benzopyrene (BaP) at sub-lethal concentrations, significant expression increase of any metacaspase gene could not be observed, expression of one metacaspase gene even decreased (Carvalho et al. 2011). High, constitutive expression of metacaspase genes during normal growth conditions has also been observed in the coccolithophore *Emiliania huxleyi* (Bidle et al. 2007) and in cryptophyte *G*. *theta* (Klemencic and Funk 2017).

Constant protein expression of metacaspases has also been reported for the green chlorophyte *Chlamydomonas reinhardtii*. *C*. *reinhardtii* contains two metacaspase genes, *CrMC1* and *CrMC2*, encoding one type I and one type II metacaspase, both with molecular mass of approximately 42 kDa. Immunodetection using an antibody directed against human caspase-3 protein revealed the constant presence of a 28-kDa protein in cells grown under normal growth conditions. Only when cells were exposed to UV-C stress (12–50 J m^−2^), the 28-kDa band gradually, but completely disappeared (Moharikar et al. 2006). However, the same antibody revealed increased levels of a ~ 17-kDa caspase-3-like epitope after treatment of *C*. *reinhardtii* cells with menadione, a quinone that undergoes redox cycles leading to the formation of superoxide radicals (Sirisha et al. 2014). Although these protein bands (28 and 17 kDa) could represent degradation products, additional experiments will be necessary to identify the caspase-3-like epitopes. Even a protein with molecular mass of approximately 12 kDa has been shown to cross-react with the caspase-3 antibody, in *Chlorella saccharophila* cells, exposed to heat shock or salt stress (Zuppini et al. 2007, 2010), while in non-treated cells, a protein band with molecular mass of approximately 34 kDa was observed, pointing again to the constant presence of a protein with caspase epitope.

### Orthocaspases in cyanobacteria

Many of the results concerning orthocaspase activity in PCD of cyanobacteria are based on enzymatic activities towards caspase substrates and, as discussed earlier, are not suitable to indicate metacaspase function. As mentioned earlier, prokaryotic orthocaspases do not recognise substrates with Asp residues at P1 position, these substrates therefore are inappropriate to determine activity of orthocaspases (Klemencic et al. 2015). Among cyanobacteria, the most extensive work on PCD was performed in the filamentous, diazotrophic genus *Trichodesmium*. *Trichodesmium erythraeum* IMS101, containing the largest reported number of orthocaspases (12 in total), also is the only cyanobacterium, in which expression levels of orthocaspases during cell death have been monitored. RNA expression of two orthocaspase genes (*TeMC1* and *TeMC9*) was investigated in cell cultures exposed to Fe starvation (Bar-Zeev et al. 2013). No expression of these genes was detected in Fe-repleted cultures, whereas increased expression levels were detected especially for *TeMC9* encoding an orthocaspase with C-terminal WD40 domains in Fe-depleted media. In another study, transcript abundance was tested for all identified *TeMC* genes 8 and 22 h after a rapid cell culture collapse in environmental samples, where more than 90% of the biomass collapsed within approximately 24 h (Spungin et al. 2016). Seven out of the twelve metacaspase genes (*TeMC1*, *TeMC3*, *TeMC4*, *TeMC7*, *TeMC8*, *TeMC9* and *TeMC11*) were found to be significantly upregulated after culture collapse, their transcripts increased up to 6.2-fold during 22 h, while no expression was detected for *TeMC12* throughout the experiment. The upregulation of metacaspase gene transcription was accompanied with increased enzymatic activity against Asp-substrates and with strong transcription decrease of genes associated with buoyancy and gas vesicle production.

The higher availability of transcriptomic data nowadays allows the general expression analysis of metacaspase genes in various cyanobacteria. The first microbial community-wide metacaspase analysis including metagenomics and metatranscriptomics was performed using samples from the brackish Baltic Sea, a water body characterised by various microenvironments and occurrences of massive cyanobacterial blooms (Asplund-Samuelsson et al. 2016). Interestingly, among all the identified microorganisms, filamentous cyanobacteria showed highest orthocaspase gene expression levels. For the three orthocaspase genes of one of the main bloom-forming cyanobacteria, *Nodularia spumigena*, a distinct seasonal expression pattern was detected accompanied by co-expression of nodularin toxin synthesis enzymes. In contrast to *Trichodesmium*, metacaspases in *N*. *spumigena* therefore seem to be involved in house-keeping functions, including PCD processes. Nevertheless, two of the three *N*. *spumigena* metacaspases (M31 and M33) belong to gene expression clusters that include several nodularin toxin synthesis genes, pointing towards a link between toxin biosynthesis, orthocaspase expression and PCD. In another bloom-forming cyanobacterium, *M*. *aeruginosa* PCC 7806, we discovered the presence of several putative genes coding for toxin-antitoxin systems, also well-known architects of prokaryotic PCD (Klemencic and Dolinar 2016). Information regarding their (co)-expression patterns will be of great importance to explain the interplay in the regulation of survival or death in cyanobacterial populations.

## Discussion

Driven by the discovery of caspase homologues in plants, fungi and prokaryotes (Uren et al. 2000), an enthusiastic research era began to confirm (not to analyse!) the involvement of orthocaspases, metacaspases and paracaspases as key players with strategic positions in programmed cell death. Accumulating evidence, however, piece by piece is revealing a much broader picture, portraying their involvement in various aspects of cellular metabolism, which often are independent of cell death.

Transcriptional analysis has shown that most orthocaspases/metacaspases in prokaryotes and algae, just like metazoan caspases, are constitutively expressed as proenzymes. Tight post-translational regulation is therefore needed to prevent undesirable proteolysis. While metazoan C14 members (caspases and paracaspases) are activated by dimerization, plant and algal type I/II/III metacaspases all strictly depend on calcium, which induces conformational changes and renders the enzymes proteolytical active (Moss et al. 2007; Watanabe and Lam 2011b). An interesting exception remain the prokaryotic orthocaspases, which were shown to specifically autoprocess upon recombinant production in *E*. *coli* without any further activation (Klemencic et al. 2015). Even though the orthocaspase MaOC1 of *M*. *aeruginosa* contains no additional C-terminal domain, cleavage of the catalytic p20 domain from the polypeptide chain was shown to be a prerequisite for its activity. As mentioned earlier, the majority of orthocaspases in cyanobacteria contain a plethora of defined domains. Since these could, based on their homology, be involved in processes like protein-protein interaction, signalling and/or chaperoning, it is possible that also the caspase p20 domain in these organisms plays a regulatory role. This would be another indication for the hypothesis that the machinery of programmed cell death evolved from a previously established toolbox, whose primary function was to regulate normal physiological cellular processes (Ameisen 2002). Only when the cell metabolisms reach beyond the point of no-return, for example, after excessive stress, actions of these proteins culminate and exhibit the apoptotic phenotype.

Investigating cyanobacterial genomes characterised up to now, we observed an interesting phenomenon in strains containing only one orthocaspase gene (for example, *Synechococcus* sp. PCC 7002, *Synechocystis* sp. PCC 6803, *M*. *aeruginosa* PCC 9806, see also Fig. 3a): this single gene encodes a protease, which most likely is catalytic inactive. Most commonly, the amino acid Tyr replaces the catalytic His and a Ser residue substitutes for the catalytic Cys (Asplund-Samuelsson et al. 2012; Jiang et al. 2010). Genes rendering proteolytic inactive orthocaspases can also be found in other cyanobacterial species or strains, which contain another one (as in *M*. *aeruginosa* PCC-9443) or more (in *N*. *punctiforme* PCC 73102) (Jiang et al. 2010) active orthocaspases. Based on expression studies, these proteolytic inactive orthocaspases seem to be important; the one gene encoding a putative proteolytic inactive orthocaspase in *M*. *aeruginosa* PCC 7806 (*MaOC2*) is the only of the six orthocaspase genes in this organism, whose expression significantly differs during the light-dark period, suggesting a role in the diurnal cycle (Straub et al. 2011). Knock-out mutants and identification of the orthocaspase binding partners should shed light on the functions of these putative proteolytic inactive proteins in cyanobacteria. However, it should also be noted that even the presence of a complete His-Cys dyad does not implicate proteolytic functionality per se and more attention should be drawn to the amino acid residues forming the specificity pocket. Substitution of Cys (Cys29) with Ala in *A*. *thaliana* AtMC9 resulted in a 90% decrease of the enzyme activity in comparison to wild type (Belenghi et al. 2007). Point mutation of the homologous Cys residue in TbMC2 (Cys92) (see Fig. 2b) also resulted in markedly reduced activity (McLuskey et al. 2012). Only in two of the *M*. *aeruginosa* orthocaspases the complete catalytic dyad as well as the conserved specificity pocket amino acids can be found: in MaOC1 of the PCC 7806 strain and in one of the two orthocaspases of the PCC 9443 strain (Fig. 3a, c), both lacking other domains beside the p20 domain. Interestingly, one of the two orthocaspases of NIES-843 contains the catalytic p20 dyad with conserved His-Cys dyad, but is lacking the Cys residue of the specificity pocket, and at the same time, it clusters together with the inactive proteases in the phylogenetic tree (Fig. 2b, we therefore marked it with parenthesis in Fig. 2a). Analysis of metacaspase-like proteins in *G*. *theta* (GtMC3-GtMC14) revealed that all GtMCs except of GtMC13 contain the catalytic dyad, but lack the conserved Cys residue of the specificity pocket. General caution should therefore be undertaken when interpreting in silico data and stating functionality of any of this type of proteins prior to their experimental validation. No proteolytic inactive caspases have been identified in Metazoa.

Despite the above-discussed variety in the family of caspases and metacaspases, all C14 members seem to be linked by some common substrates. A conserved TSN protein (Tudor staphylococcal nuclease) was shown to be cleaved by the mammalian caspase-3 as well as the type II metacaspase from *Pica abies*, mcII-Pa (Sundstrom et al. 2009). Poly (ADP-ribose) polymerase (PARP), a known substrate of caspase-3 during apoptosis, has also been shown to be proteolysed by the yeast metacaspase Yca1 during PCD in fungi (Strobel and Osiewacz 2013). This metacaspase was further demonstrated to cleave GAPDH, glyceraldehyde 3-phosphate dehydrogenase, a caspase-1 substrate, in an NO-dependent manner (Silva et al. 2011). Proteins of various functional categories were identified as AtMC9 substrates in a proteomic study (Tsiatsiani et al. 2013) including actin, ribosomal proteins, as well as proteins belonging to chaperone families (Tsiatsiani et al. 2011).

The available data on PCD or cellular maintenance in chloroplast-containing organisms often are conflicting regarding the presence or involvement of C14 members. Obviously, our knowledge still is scarce, preventing us to answer the question whether orthocaspases and metacaspase-like proteases are involved in unicellular PCD. Research in the field of ortho−/metacaspases should unbiasedly approach the role of these fascinating enzymes, rather than imposing populistic statements on their function. Targeting the specific metacaspase genes, as well as their in vivo detection should be prioritised before using generally available kits, which are based on caspase activity (Salvesen et al. 2016). Proteases, including caspases, paracaspases, orthocaspases and metacaspases are undoubtedly involved in a plethora of cellular processes and whole-cell metagenomic, transcriptomic and proteomic approaches would therefore not only give information about the proteases of interest, but could reveal their interaction partners, shed light on their native substrates and therefore reveal a broader perspective of their involvement in the cellular environment.

## Electronic supplementary material

Online resource 1 (PDF 119 kb)
