# Supplementary material for: Structural and functional diversity of caspase homologues in non-metazoan organisms
Source: Protoplasma. 2017 Jul 25;255(1):387–97. doi: 10.1007/s00709-017-1145-5 (PMC5756287; doi:10.1007/s00709-017-1145-5)
Supplement: Supplementary file 1 — (PDF 119 kb) [file 709_2017_1145_MOESM1_ESM.pdf]

Online resource 1

Structural and functional diversity of caspase homologues in non-metazoan organisms

Marina Klemenčič[1,2] and Christiane Funk[1]

[1]Department of Chemistry, Umeå University, SE-901 87, Umeå, Sweden

[2]Department of Chemistry and Biochemistry, Faculty of Chemistry and Chemical Technology, University of Ljubljana, Večna pot 113, SI-1000 Ljubljana, Slovenia

Corresponding author: Marina Klemenčič, marina.klemencic@umu.se

Table S1 NCBI identifiers for all orthocaspases used in phylogenetic study in Fig. 3

Name	NCBI Identifier
**PCC7806_1**	CAO88281
**PCC7806_2**	CAO87907
**PCC7806_3**	CAO88826
**PCC7806_4**	CAO87362
**PCC7806_5**	CAO86659
**PCC7806_6**	CAO86810
**NIES843_1**	WP_012265613
**NIES843_2**	WP_012265065
**PCC 9808_1**	WP_002793052
**PCC 9808_2**	CCI21641
**PCC 9807_1**	WP_002789827
**PCC 9807_2**	WP_002788983
**PCC 9717_1**	WP_002758885
**PCC 9717_2**	CCH95863.1
**PCC 9717_3**	WP_002759524
**PCC 9443_1**	WP_002765082
**PCC 9443_2**	WP_002768321
**PCC 9432_1**	WP_004159143
**PCC 9432_2**	WP_002753269
**PCC 9432_3**	WP_004158025
**PCC9432_4**	WP_002754967
**PCC9809_1**	WP_002798807
**PCC9809_2**	WP_002795796
**PCC7941_1**	WP_002773629
**PCC7941_2**	WP_002776611
**PCC7941_3**	WP_002775431
**PCC9701_1**	CCI37999
**PCC9701_2**	WP_002802945
**PCC9701_3**	WP_002800316
**PCC9806**	WP_002785011
**NIES44**	GAL95464
**TAIHU98_1**	ELP52061
**TAIHU98_2**	WP_042791100
**TAIHU98_3**	ELP56295
**T1-4**	WP_008206103
**NIES2549**	WP_046661785
